# Extracellular acidification-induced CXCL8 production through a proton-sensing receptor OGR1 in human airway smooth muscle cells: a response inhibited by dexamethasone

**DOI:** 10.1186/s12950-019-0207-1

**Published:** 2019-02-19

**Authors:** Maiko Kadowaki, Hidenori Yamada, Koichi Sato, Hiroko Shigemi, Yukihiro Umeda, Miwa Morikawa, Yuko Waseda, Masaki Anzai, Yosuke Kamide, Haruka Aoki-Saito, Takeshi Hisada, Fumikazu Okajima, Tamotsu Ishizuka

**Affiliations:** 1 Third Department of Internal Medicine, Faculty of Medical Sciences, University of Fukui, 23-3 Matsuoka-Shimoaizuki, Eiheiji, Fukui, 910-1193 Japan; 2 Department of Medicine and Molecular Science, Gunma University Graduate School of Medicine, 3-39-15 Showa-machi, Maebeshi, 371-8511 Japan; 3 Laboratory of Signal Transduction, Institute for Molecular and Cellular Regulation, Gunma University, 3-39-15 Showa-machi, Maebeshi, 371-8511 Japan; 4 Laboratory of Signal Transduction, Faculty of Pharmaceutical Sciences, Aomori University, 2-3-1 Kobata, Aomori, 030-0943 Japan

**Keywords:** OGR1, Airway smooth muscle cell, Proton, CXCL8, NF-κB, Dexamethasone, Bronchial asthma, COPD

## Abstract

**Background:**

Human airway smooth muscle cells (ASMCs) contribute to bronchial contraction and airway hyperresponsiveness in patients with bronchial asthma. They also generate cytokines, chemokines, and matricellular proteins. Ovarian cancer G protein-coupled receptor 1 (OGR1) senses extracellular protons and mediates the production of interleukin-6 (IL-6) and connective tissue growth factor (CTGF) in ASMCs.

**Methods:**

ASMCs were stimulated for the indicated time by pH 6.3 or pH 7.4-adjusted Dulbecco’s Modified Eagle Medium (DMEM) containing 0.1% bovine serum albumin (BSA) (0.1% BSA-DMEM). As a control stimulant, pH 7.4-adjusted 0.1% BSA-DMEM containing 10 ng/mL tumor necrosis factor-α (TNF-α) was used. Interleukin-8/C-X-C motif chemokine ligand 8 (CXCL8) mRNA expression in ASMCs was quantified by RT-PCR using real-time TaqMan technology. CXCL8 secreted from ASMCs was measured by enzyme-linked immunosorbent assay (ELISA). Phosphorylation at serine 536 of NF-κB p65 and binding of p65 to oligonucleotide containing an NF-κB consensus binding site were analyzed by Western blotting and an ELISA-based kit.

**Results:**

Acidic pH induced a significant increase of CXCL8 mRNA expression and CXCL8 protein secretion in ASMCs. ASMCs transfected with small interfering RNA (siRNA) targeted for OGR1 produced less CXCL8 compared with those transfected with non-targeting siRNA. Protein kinase C (PKC) inhibitor, MEK1/2 inhibitor, and the inhibitor of IκB phosphorylation reduced acidic pH-stimulated CXCL8 production in ASMCs. Dexamethasone also inhibited acidic pH-stimulated CXCL8 production of ASMCs in a dose-dependent manner. Dexamethasone did not affect either phosphorylation or binding to the consensus DNA site of NF-κB p65.

**Conclusions:**

CXCL8 released from ASMCs by extracellular acidification may play a pivotal role in airway accumulation of neutrophils. Glucocorticoids inhibit acidic pH-stimulated CXCL8 production independent of serine 536 phosphorylation and the binding to DNA of NF-κB p65, although NF-κB activity is essential for CXCL8 production in ASMCs.

## Background

Bronchial asthma is a disease characterized by chronic airway inflammation, airway hyperresponsiveness, and reversible airway obstruction. Airway smooth muscle cell hyperplasia and hypertrophy are often observed in patients with severe asthma. An increase in airway smooth muscle mass could contribute to airway obstruction and hyperresponsiveness [1]. Human airway smooth muscle cells (ASMCs) may play an important role in airway inflammation because they can release cytokines such as IL-6 and CXCL8 in response to environmental stimuli [2–8]. Extracellular pH is strictly maintained at 7.35 to 7.45 in the blood. Although the precise mechanism of the acid-base balance in the airway is not fully understood, airway acidification is a well-recognized feature in asthma and contributes to the pathophysiology of the disease [9, 10]. The degree of acidification depends on the severity of asthma, and airway pH seems to reach 5.2. It is normalized by corticosteroid therapy [9, 11].

Previous studies have shown that the proton-sensing capsaicin-sensitive TRPV1 channel and acid-sensing ion channels (ASICs) in sensory nerves play critical roles in asthmatic symptoms [10, 12, 13]. It has also been demonstrated that ovarian cancer G protein-coupled receptor 1 (OGR1) family G protein-coupled receptors (GPCRs), including OGR1 (also known as GPR68), G protein-coupled receptor 4 (GPR4), and T-cell death-associated gene 8 (TDAG8 or GPR65), sense extracellular protons and mediate cellular actions induced by alkaline and acidic pHs of 8 to 6 through histidine residues in a variety of cell types [14–17]. We have shown that, among these proton-sensing GPCRs, only OGR1 is expressed in ASMCs, and that IL-6 and CTGF are secreted from ASMCs through OGR1-mediated stimulation of intracellular signaling pathways in response to extracellular acidification [18, 19]. Interleukin-8 (IL-8/CXCL8) is a member of the CXC chemokine subfamily of cytokines and a potent neutrophil chemoattractant. Because the most results of clinical trials of anti-CXCL8 or C-X-C motif chemokine receptor 2 (CXCR2) antagonists in asthma or chronic obstructive pulmonary disease (COPD) are disappointing, these bring into question the role of CXCL8-mediated neutrophil recruitment in the pathogenesis of asthma and COPD [20, 21]. However, elevated concentrations of CXCL8 are found in sputum, bronchoalveolar lavage fluid, and bronchial tissue of patients with respiratory diseases such as severe asthma, occupational asthma, cystic fibrosis, and COPD [22, 23]. CXCL8 is secreted by different cell types including alveolar macrophages [24], airway epithelial cells [25], and ASMCs [7, 26–34] following stimulation by viruses, various cytokines, hypoxia, reactive oxygen species, and bacterial particles.

In the current study, extracellular acidification was shown to increase CXCL8 secretion from ASMCs through OGR1-mediated stimulation, and this secretion was inhibited by dexamethasone (DEX) in a dose-dependent manner. The intracellular cell signaling and NF-κB activation involved in acidic pH-induced CXCL8 production were explored, and the effects of DEX on serine 536 phosphorylation and DNA binding of NF-κB p65 in ASMCs were examined.

## Methods

### Materials and reagents

Recombinant human tumor necrosis-α (TNF-α) was from Peprotech (Rocky Hill, NJ). U0126, SB-203580, and bisindolylmaleimide I were from Cayman Chemical (Ann Arbor, MI). BAY 11–7082 was from Focus Biomolecules (Plymouth Meeting, PA). Goat anti-rabbit IgG-HRP was from GE Healthcare Japan Corporation (Tokyo, Japan). Antibodies for phospho-NF-κB p65 (Ser536) (93H1) and glyceraldehyde-3-phosphate dehydrogenase (GAPDH) (14C10) were from Cell Signaling Technology (Danvers, MA). Fatty acid-free bovine serum albumin (BSA) was from EMD chemicals (San Diego, CA). Dexamethasone (DEX), Dulbecco’s Modified Eagle Medium (DMEM), and other chemicals were purchased from Sigma-Aldrich (St. Louis, MO).

### Cell culture

Human bronchial smooth muscle cells (BSMCs, catalog No. CC-2576) originated from non-diseased individuals were purchased from Lonza (Walkersville, MD). Eight to nine passages of BSMCs were used as human airway smooth muscle cells (ASMCs) in this study. Cells were grown in the complete culture medium (Lonza): smooth muscle-cell basal medium supplemented with human epidermal growth factor, insulin, human fibroblast growth factor β, 5% fetal bovine serum, gentamicin, and amphotericin B, under a humidified atmosphere of 95% air plus 5% CO_2_ at 37 °C, as previously reported [19]. ASMCs were cultured in 12-well plates for ELISA of CXCL8, 6-well plates for real-time PCR using TaqMan probes or Western blotting, and 10-cm culture dishes for extraction of nuclear protein.

### Cell stimulation and ELISA of human CXCL8

The complete culture medium of ASMCs in 12-well plates was switched to DMEM containing 0.1% BSA (0.1% BSA-DMEM) before 16 h in each experiment. ASMCs were stimulated by switching the medium to pH-adjusted 0.1% BSA-DMEM (pH 6.3, 6.6, 7.0, 7.2 or 7.4) or 10 ng/mL TNF-α (pH 6.3, 7.0, or 7.4) and incubated for the indicated time. The pH of the DMEM containing 25 mM HEPES, 27 mM NaHCO_3_, and 0.1% BSA was adjusted by titration with HCl or NaOH. Inhibitors of cell signaling molecules and DEX were added 30 min before stimulation. Supernatants of cells were collected and stored at − 30 °C. Concentrations of CXCL8 were measure by enzyme-linked immunosorbent assay (ELISA) (Duo Set® ELISA development system, R&D Systems, Minneapolis, MN).

### Quantitative RT-PCR using real-time TaqMan technology

Total RNA was isolated using the RNeasy^Ⓡ^ Plus Mini Kit (Qiagen, Hilden, Germany) according to the manufacturer’s instructions. Up to 2.5 μg of total RNA were reverse-transcribed using random priming and reverse transcriptase (SuperScript™ VILO™ Master Mix, Thermo Fisher Scientific, Waltham, MA). To evaluate the expression levels of OGR1, CXCL8, and glyceraldehyde-3-phosphate dehydrogenase (GAPDH) mRNA, quantitative RT-PCR was performed using real-time TaqMan technology with a StepOne™ real-time PCR system (Thermo Fisher Scientific). TaqMan probes specific for human OGR1 (Hs00268858_s1), CXCL8 (Hs00174103_m1), and GAPDH (Hs02758991_g1) were purchased from Thermo Fisher Scientific. The expression levels of CXCL8 or OGR1 mRNA were normalized to the relative ratio of the expression of GAPDH mRNA, as previously reported [18, 19].

### Transfection of small interfering RNA

Small interfering RNA (siRNA) targeted for OGR1 (OGR1-siRNA) and non-targeting siRNA (NT-siRNA) as a control were purchased from Thermo Fisher Scientific. The ID number was L-005591-00-0005 for OGR1-siRNA and D-001810-10-05 for NT-siRNA. OGR1-siRNA or NT-siRNA was transfected at a final concentration of 3 nM into cells using RNAiMAX reagent (Thermo Fisher Scientific) according to the manufacturer’s instructions. After ASMCs suspended in the complete culture medium without gentamicin and amphotericin B were mixed with siRNA and the RNAiMAX reagent, they were then cultured in 6 or 12-well plates for 48 h before using them in further experiments.

### Western blotting

The incubation was terminated by washing once with ice-cold PBS and adding 0.1 ml of the lysis buffer composed of 50 mM Tris (pH 8.0), 150 mM NaCl_2_, 0.5% sodium deoxycholate, 0.1% sodium dodecyl sulfate, 1% NP-40 substitute, 1.04 mM AEBSF, 0.8 μM aprotinin, 0.04 mM bestatin, 14 μM E-64, 20 μM leupeptin, and 15 μM pepstatin A. The cells were then harvested from the dishes with cell scrapers. The recovered lysate was incubated for 30 min on ice and centrifuged at 17,800 *g* for 15 min. The supernatant was then analyzed by Western blotting with specific antibodies for phospho-NF-κB p65 (Ser 536) and GAPDH.

### NF-κB p65 transcription factor assay

ASMCs were incubated with 1 μM DEX or control vehicle, 0.1% ethanol (EtOH), for 30 min and stimulated by replacing the medium to 0.1% BSA-DMEM containing 10 ng/mL TNF-α (pH 7.4), 0.1% BSA-DMEM (pH 7.4), or 0.1% BSA-DMEM (pH 6.3). Nuclear protein was extracted at 60 min after each stimulation using a nuclear extract kit (Active Motif, Carlsbad, CA). Activation of NF-κB p65 was measured using the TransAM® NFκB family transcription factor assay kit (Active Motif) according to the manufacturer’s instructions. Briefly, nuclear extracts were applied to a 96-well plate to which an oligonucleotide containing the NF-κB consensus site (5’-GGGACTTTCC-3′) had been immobilized. The active form of NF-κB contained in nuclear extracts specifically bound to this oligonucleotide was detected and quantified using an anti-p65 specific antibody. DNA binding of NF-κB p65 was measured as OD 450 nm.

### Statistical analysis

All experiments were performed independently at least three times. The results of multiple observations are expressed as means ± SEM. The data were analyzed using Excel statistics software (SSRI, Tokyo, Japan). Differences between the mean values of two independent groups were determined using Student’s t-test. Paired t test was used to analyze a statistical difference between two conditions. In analyses of more than two groups, ANOVA was used to examine the significance of differences, and post hoc analysis (Bonferroni test) was performed when significance was found. *P* values less than 0.05 were considered significant.

## Results

### Extracellular acidification increases CXCL8 production of ASMCs

**Fig. 1:**
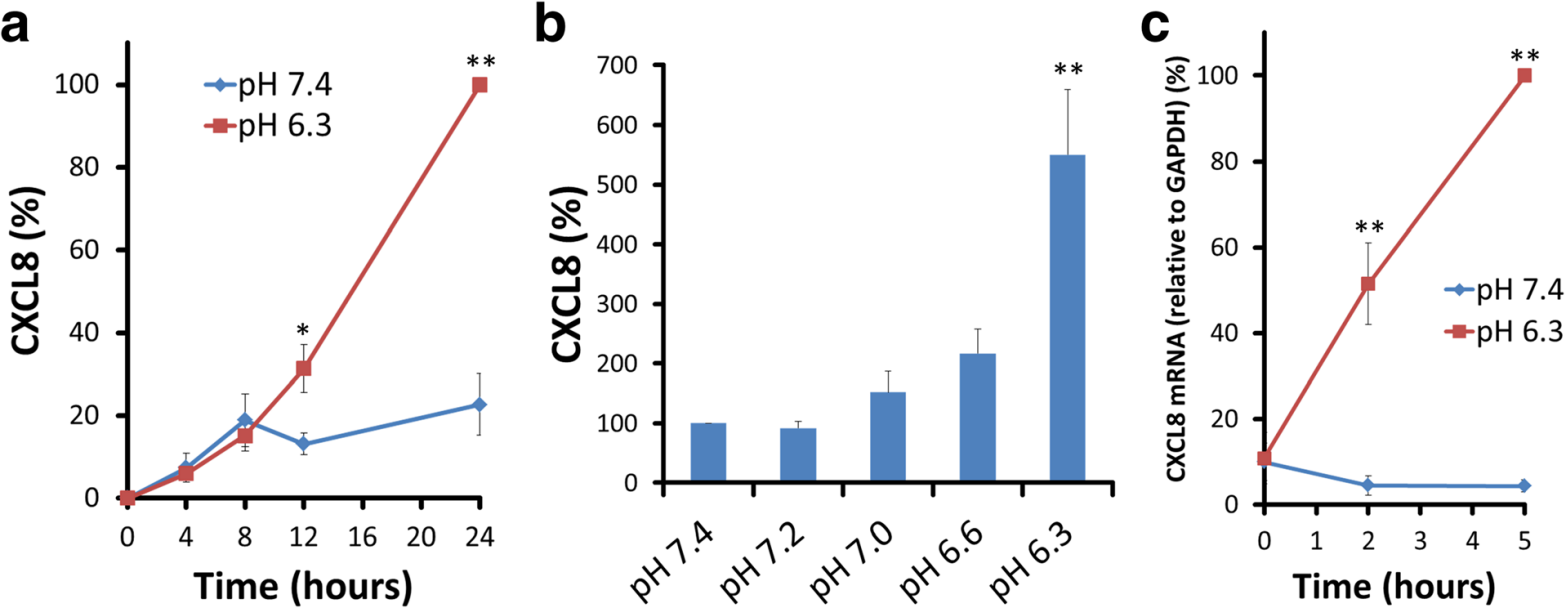
Acidic pH-induced CXCL8 protein secretion and mRNA expression in ASMC. **a** CXCL8 is produced by extracellular acidification in ASMCs. ASMCs generate CXCL8 protein by extracellular acidification in a time-dependent manner. Data are expressed as percentages of CXCL8 values obtained at pH 6.3 for 24 h (mean ± SEM, *n* = 3; **, *P* < 0.01; *, *P* < 0.05 compared with pH 7.4). **b** CXCL8 secretion is significantly increased at pH 6.3 compared with that at pH 7.4. Data are expressed as percentages of CXCL8 values obtained at pH 7.4 for 24 h (mean ± SEM, *n* = 4; **, *P* < 0.01). **c** CXCL8 mRNA expression is increased until 5 h after pH 6.3 stimulation. The expression levels of CXCL8 mRNA are standardized to those of GAPDH mRNA. Data are expressed as percentages of CXCL8 mRNA expression at 5 h after pH 6.3-stimulation (mean ± SEM, *n* = 3; **, *P* < 0.01)

**Fig. 2:**
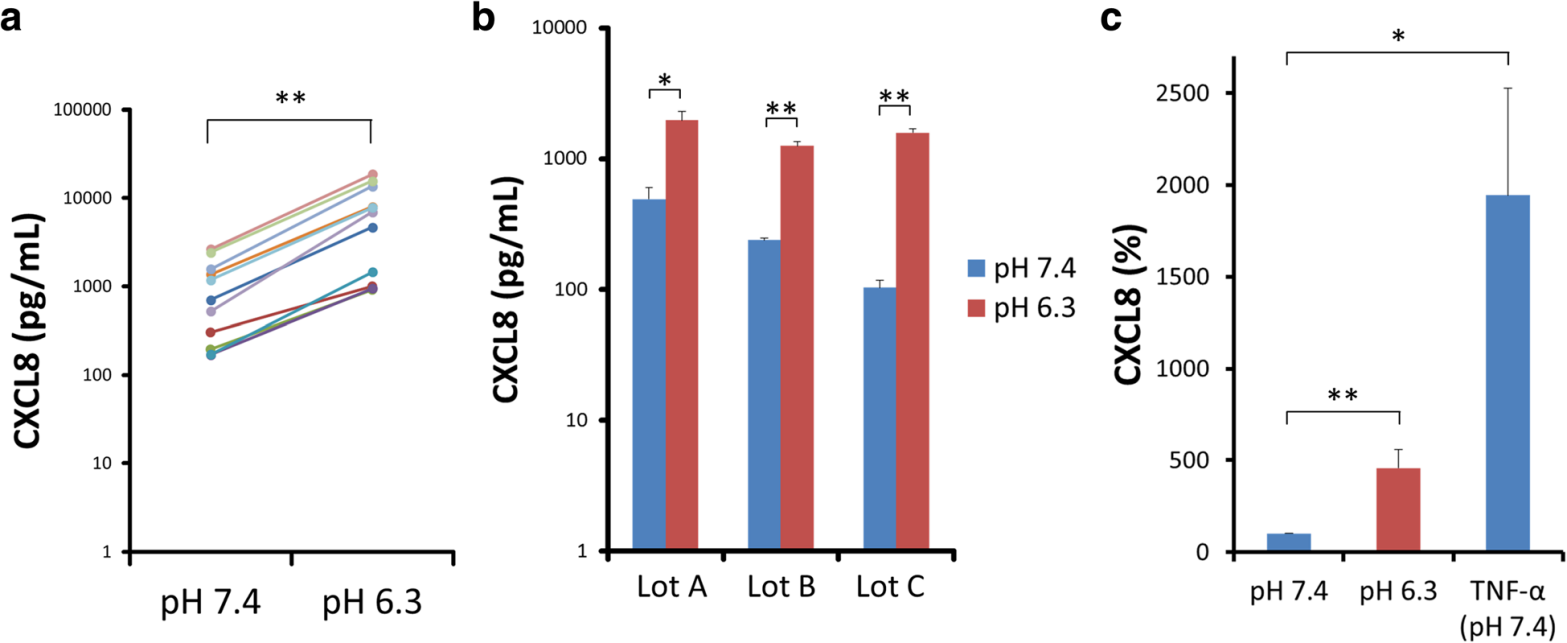
Acidic pH or TNF-α-stimulated CXCL8 secretion in ASMCs **a** The absolute values of CXCL8 secreted at pH 6.3 and 7.4 in the lot of ASMCs mainly used in this study are shown. In all independent experiments, extracellular pH 6.3 increases CXCL8 secretion at 24 h in ASMCs compared with pH 7.4 (*n* = 11, **; *P* < 0.01). **b** Extracellular acidification (pH 6.3) induces significant increase of CXCL8 secretion at 24 h compared with pH 7.4 in other three kinds of ASMCs, lot A, B and C, originated from different non-diseased individuals (mean ± SEM, *n* = 4; **, *P* < 0.01; *, *P* < 0.05). **c** The amount of CXCL8 generated from ASMCs at 24 h after pH 6.3-stimulation is 23.5% of that generated from TNF-α (10 ng/mL)-stimulated ASMCs. Data are expressed as percentages of CXCL8 values obtained at pH 7.4 without TNF-α for 24 h (mean ± SEM, *n* = 4; **, *P* < 0.01; *, *P* < 0.05)

Whether extracellular acidification affected CXCL8 mRNA and protein expression was examined first. ASMCs were serum deprived for 16 h in 0.1% BSA-DMEM and then stimulated by replacing with pH 6.3-adjusted 0.1% BSA-DMEM or pH 7.4-adjusted 0.1% BSA-DMEM. The cell supernatants were obtained at 4, 8, 12, and 24 h after stimulation. Acidic pH (pH 6.3) induced significantly more production of CXCL8 protein than pH 7.4. Although CXCL8 production was observed in incubation with pH 7.4-adjusted medium, it was increased about 5-fold in incubation with pH 6.3-adjusted medium compared with pH 7.4 at 24 h (Fig. 1a). We examined the effects of various extracellular pH on CXCL8 secretion in ASMC. Acidic pH (less than pH 7.0) seemed to increase CXCL8 secretion. Significant increase of CXCL8 secretion compared with extracellular pH 7.4 was observed at pH 6.3 (Fig. 1b). To examine the mRNA expression of CXCL8, ASMCs were incubated for 2 or 5 h in pH 6.3- or pH 7.4-adjusted medium. CXCL8 mRNA was significantly increased at 2 or 5 h after replacing with pH 6.3-adjusted medium compared with pH 7.4-adjusted medium (Fig. 1c). The absolute values of CXCL8 secreted from ASMSs for 24 h at pH 6.3 and 7.4 in 19 independent experiments are shown in Fig. 2a. The mean CXCL8 secreted from ASMSs for 24 h in pH 7.4 adjusted medium was 1.0 ng/mL. The mean CXCL8 generated at 24 h after pH 6.3-stimulation was 7.2 ng/mL. We mainly used this lot of ASMCs originated from a non-diseased individual in our study. In order to make sure that the acidic-pH induced CXCL8 secretion is a common feature of ASMCs, we investigated acidic pH-stimulated CXCL8 secretion in other three kinds of ASMCs (lot A, B, and C) originated from non-diseased individuals. Although the amount of CXCL8 secreted at pH 7.4 and pH 6.3 in each lot of ASMCs was various, acidic pH (pH 6.3) induced significant increases of CXCL8 secretion in all lots of ASMCs (Fig. 2b). The amount of CXCL8 secreted by pH 6.3-stimulation was about 20–25% of CXCL8 generated from TNF-α (10 ng/mL)-stimulated ASMCs (Fig. 2c).

### OGR1 is involved in acidic pH-stimulated CXCL8 production in ASMCs

**Fig. 3:**
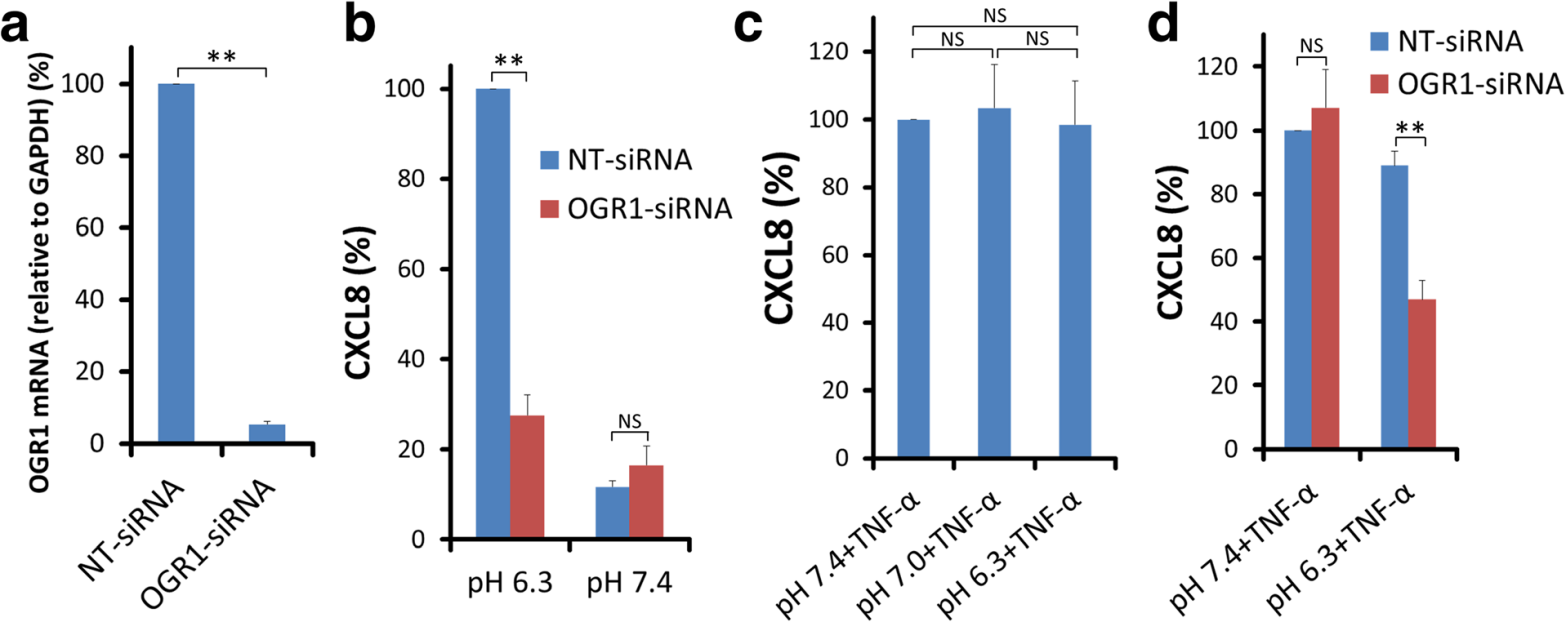
Possible involvement of OGR1 in CXCL8 production of ASMCs**. a** ASMCs are transfected with non-targeting RNA (NT-siRNA) or siRNA specific for OGR1 (OGR1-siRNA). The expression of OGR1 mRNA was measured by real-time TaqMan RT-PCR at 48 h after transfection. The expression levels of OGR1 mRNA are standardized to those of GAPDH mRNA expression, and data are expressed as percentages of OGR1 mRNA expression in ASMCs transfected with NT-siRNA (mean ± SEM, *n* = 5; **, *P* < 0.01). **b** At 48 h after ASMCs were transfected with either NT-siRNA or OGR1-siRNA, ASMCs were incubated in pH 6.3 or 7.4 medium for an additional 24 h. Acidic pH-stimulated CXCL8 secretion is suppressed in ASMCs transfected with OGR1-siRNA compared with those transfected with NT-siRNA. Data are expressed as percentages of CXCL8 values in the supernatants of ASMCs transfected with NT-OGR1 after 24-h stimulation by pH 6.3 medium (mean ± SEM, *n* = 3; **, *P* < 0.01; NS, not significant). **c** CXCL8 secretion in TNF-α (10 ng/mL)-stimulated ASMCs is not influenced by extracellular pH (pH 6.3, pH 7.0, and pH 7.4). Data are expressed as percentages of CXCL8 values obtained from the ASMCs stimulated by TNF-α (10 ng/mL) at pH 7.4 for 24 h (mean ± SEM, *n* = 4; NS, not significant). **d** TNF-α (10 ng/mL)-stimulated CXCL8 secretion is not inhibited by OGR1 siRNA at pH 7.4, although it is significantly inhibited at pH 6.3. Data are expressed as percentages of CXCL8 values in the supernatants of ASMCs transfected with NT-OGR1 after 24 h-stimulation of TNF-α at pH 7.4 (mean ± SEM, *n* = 8; **, *P* < 0.01; NS, not significant)

In our previous investigation, only OGR1 mRNA was practically detected in ASMCs among four kinds of proton-sensing GPCRs, OGR1, GPR4, TDAG8, and G2A. The expression level of OGR1 mRNA after treatment with OGR1-siRNA was decreased to about 5% of the level found after treatment with NT-siRNA in ASMCs (Fig. 3a). The amount of CXCL8 protein generated by extracellular acidification was significantly deceased in ASMCs transfected with OGR1-siRNA compared with that in ASMCs transfected with NT-siRNA (Fig. 3b). Acidic pH (pH 6.3) as well as pH 7.0 did not enhance CXCL8 secretion in TNF-α (10 ng/mL)-stimulated ASMCs compared with pH 7.4. Namely, additive effects of acidic pH and TNF-α were not observed at all (Fig. 3c). This result was different from the phenomenon which we observed in transforming growth factor (TGF)-β (1 ng/mL)-stimulated ASMCs because acidic pH (pH 6.3) enhanced TGF-β-stimulated CTGF secretion in ASMCs [19]. We examined the effects of OGR1 siRNA transfection on TNF-α-stimulated CXCL8 secretion in ASMCs. TNF-α (10 ng/mL)-stimulated CXCL8 secretion was not inhibited by OGR1 siRNA at pH 7.4, suggesting that OGR1 siRNA did not inhibit CXCL8 secretion in ASMCs nonspecifically. However, it also inhibited TNF-α (10 ng/mL)-stimulated CXCL8 secretion at pH 6.3, although the detailed mechanism of this inhibition is unclear (Fig. 3d).

### Possible involvements of protein kinase C (PKC), extracellular signal-regulated kinase (ERK), and NF-κB in extracellular acidification-induced CXCL8 production of ASMCs

**Fig. 4:**
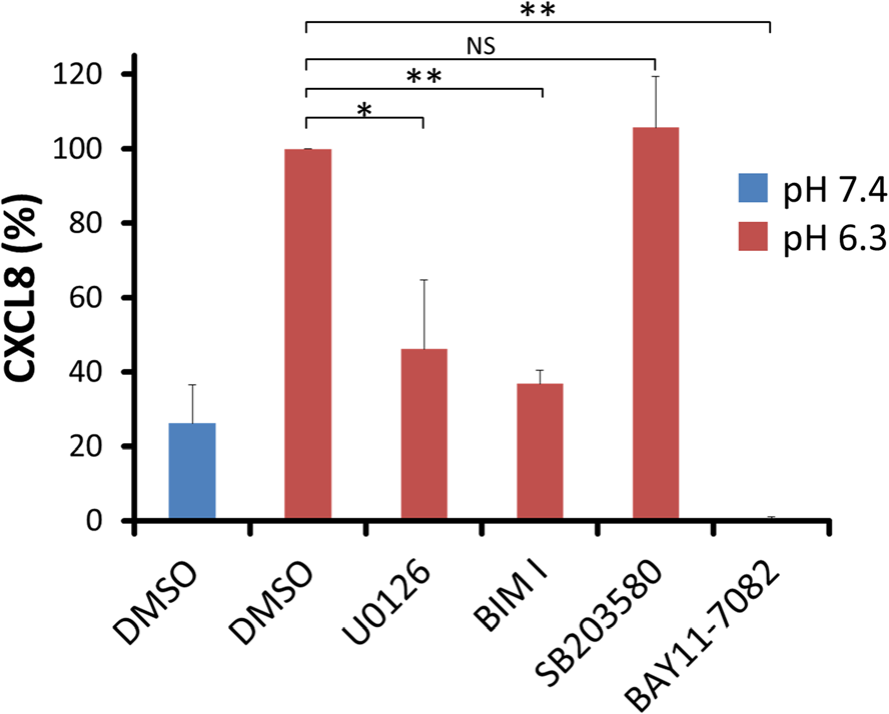
Possible involvements of PKC, ERK1/2, p38 MAPK, and NF-κB in CXCL8 production of acidic pH-stimulated ASMCs. ASMCs were pretreated with 10 μM bisindolylmaleimide I (BIM I), 10 μM U0126, 10 μM SB203580, 30 μM BAY11–7082, or control vehicle (0.1% DMSO) for 30 min. The cells were further incubated at the indicated pH with 10 μM BIM I, 10 μM U0126, 10 μM SB203580, 30 μM BAY11–7082, or control vehicle (0.1% DMSO) for an additional 24 h. CXCL8 secretion induced by extracellular acidification in ASMCs is significantly suppressed by U0126 and BIM I, but not SB203580. BAY11–7082 almost completely inhibits CXCL8 secretion in ASMCs. Data are expressed as percentages of CXCL8 values in the supernatants of pH 6.3-stimulated ASMCs without any inhibitors. The data are expressed as means ± SEM (*n* = 4, **, *P* < 0.01; *, *P* < 0.05; NS, not significant)

We previously showed that ERK1/2 and p38 mitogen-activated protein kinase (p38 MAPK) were phosphorylated and activated by acidic pH in ASMCs [18]. The effects of PKC inhibitor bisindolylmaleimide I (BIM I), MEK1/2 inhibitor U0126, and p38 MAPK inhibitor SB203580 on acidic pH-stimulated CXCL8 production were examined. As control vehicle, 0.1% dimethyl sulfoxide (DMSO) was used. BIM I and U0126, but not SB203580, significantly inhibited acidic pH-stimulated CXCL8 production. BAY 11–7082 is an irreversible inhibitor of IκB kinase α (IKKα) and phosphorylation of IκBα. It is known that it irreversibly inhibits TNF-α-induced IκBα phosphorylation, resulting in the inactivation of NF-κB [35]. BAY11–7082 completely blocked pH 6.3-stimulated CXCL8 production. The amount of CXCL8 generated from acidic pH-stimulated ASMCs in the presence of BAY11–7082 was significantly less than that generated from ASMCs without acidic pH stimulation (Fig. 4).

### Effects of dexamethasone on CXCL8 production in ASMCs stimulated by extracellular acidification

**Fig. 5:**
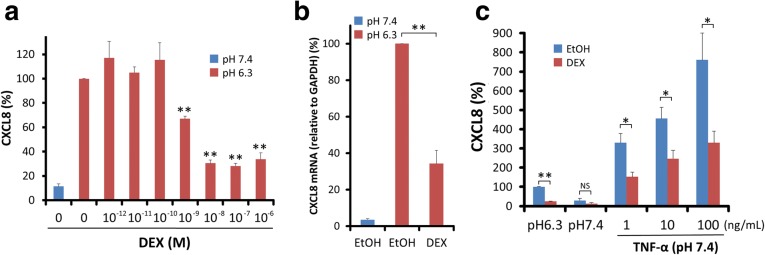
Effects of dexamethasone (DEX) on CXCL8 production in acidic pH or TNF-α-stimulated ASMCs. **a** ASMCs were pretreated with DEX (10^− 12^-10^− 6^ M) or control vehicle (0.1% ethanol) for 30 min. The cells were further incubated at the indicated pH with DEX or control vehicle for 24 h. DEX inhibits CXCL8 production of acidic pH-stimulated ASMCs in a dose-dependent manner. Its inhibition is significant at 1 nM and over of DEX. Data are expressed as percentages of CXCL8 values in the supernatants of pH 6.3-stimulated ASMCs without DEX (mean ± SEM, *n* = 4; **, *P* < 0.01). **b** DEX (1 μM) significantly inhibits CXCL8 mRNA expression in ASMCs at 5 h after acidic pH-stimulation. The expression levels of CXCL8 mRNA are standardized to those of GAPDH mRNA. Data are expressed as percentages of CXCL8 mRNA expression at 5 h after pH 6.3-stimulation (mean ± SEM, *n* = 3; **, *P* < 0.01). **c** TNF-α (1–100 ng/mL) induces CXCL8 secretion for 24 h in a dose-dependent manner. DEX (1 μM) partially but significantly inhibits CXCL8 production of TNF-α-stimulated ASMCs in each concentration of TNF-α. The inhibition rate of CXCL8 secretion by DEX in TNF-α-stimulated ASMCs seems to be smaller than that in acidic pH-stimulated ASMCs. Data are expressed as percentages of CXCL8 values of pH 6.3-stimulated ASMCs without DEX (mean ± SEM, *n* = 4; **, *p* < 0.01; *, *P* < 0.05; NS, not significant)

It is well known that CXCL8 production induced by TNF-α is inhibited by glucocorticoids in many kinds of cells [26, 27, 36–38]. The effects of dexamethasone (DEX) (10^− 12^–10^− 6^ M) on acidic pH or TNF-α-stimulated CXCL8 production were examined in ASMCs. DEX inhibited acidic pH-stimulated CXCL8 production in ASMCs in a dose-dependent manner. The inhibition was significant at 1 nM and over of DEX (Fig. 5a). DEX (1 μM) also significantly inhibited CXCL8 mRNA expression in pH 6.3-stimulated ASMCs (Fig. 5b). We examined dose-dependent effects of TNF-α on CXCL8 secretion in ASMCs. TNF-α induced CXCL8 secretion in a dose-dependent manner at 1–100 ng/mL. DEX (1 μM) partially but significantly inhibited TNF-α (1–100 ng/mL)-stimulated CXCL8 production in ASMCs. The inhibition rate of DEX on TNF-α -stimulated CXCL8 secretion was about 50% (Fig. 5c).

### Effects of DEX on extracellular acidification-induced phosphorylation of NF-κB p65 and DNA binding of NF-κB p65

**Fig. 6:**
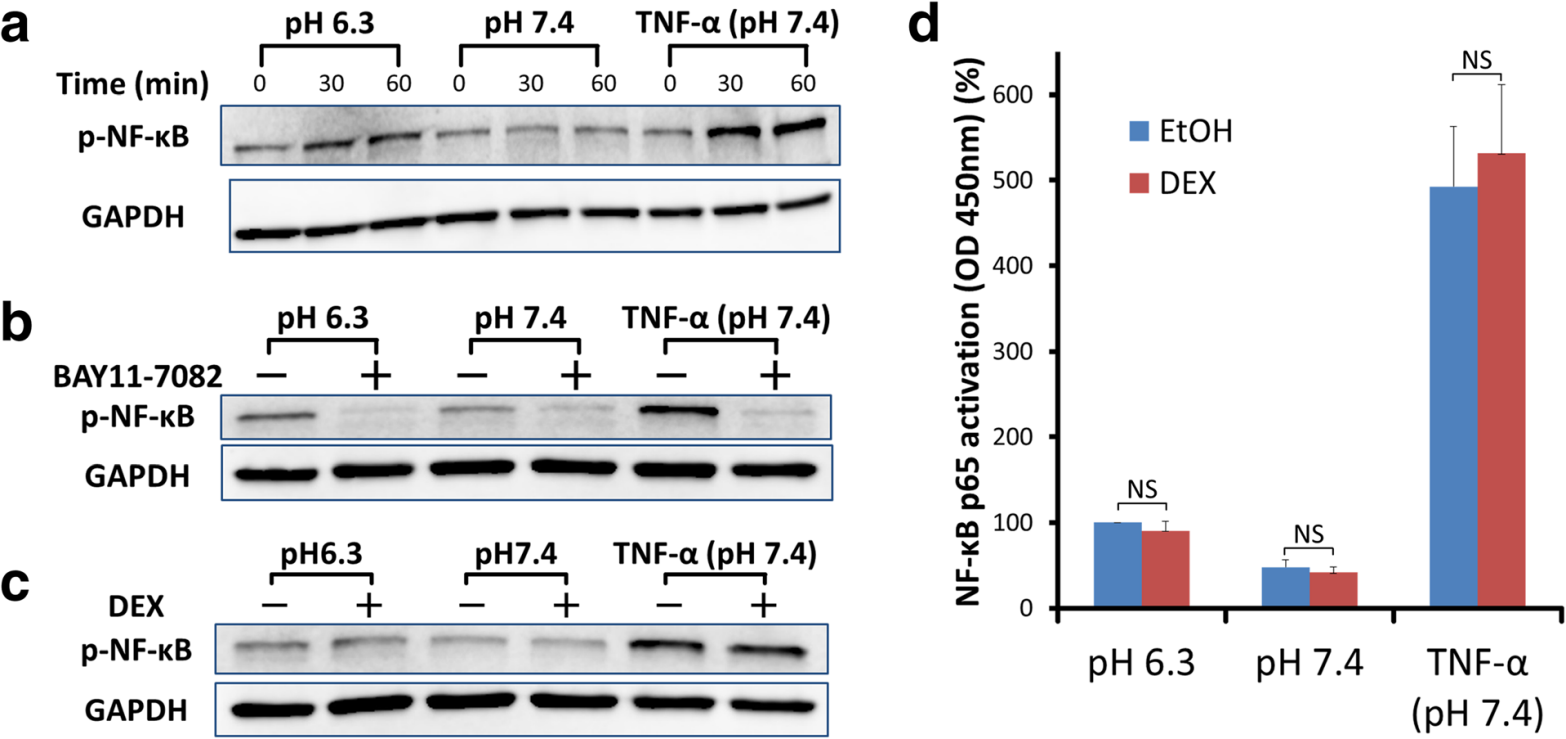
Effects of DEX on phosphorylation and DNA binding of NF-κB p65. **a** ASMCs were incubated for the indicated time in pH 6.3 or pH 7.4-adjusted medium. As a positive control of serine 536 phosphorylation and DNA binding of NF-κB p65, ASMCs were stimulated with 10 ng/ml TNF-α in the pH 7.4-adjusted medium. A weak increase of phospho-NF-κB p65 (Ser 536) is observed in ASMCs at 30–60 min after pH 6.3-stimulation, but not after pH 7.4-stimulation. GAPDH was used as an endogenous control protein. Representative data of 3 independent experiments are shown. **b** ASMCs were pretreated with 30 μM BAY11–7082 or control vehicle (0.1% DMSO) for 30 min. The cells were further incubated for 60 min in the pH 6.3-adjusted medium, pH 7.4-adjusted medium, or pH 7.4-adjusted medium containing 10 ng/ml TNF-α. BAY11–7082 almost completely decreases the expressions of phospho-NF-κB p65 (Ser536) in all cases, pH 6.3, pH 7.4, and TNF-α (pH 7.4). Representative data of 3 independent experiments are shown. **c** After 30-min preincubation with DEX (1 μM) or control vehicle (0.1% ethanol), ASMCs were stimulated for 60 min by pH 6.3-adjusted medium, pH 7.4-adjusted medium, or pH 7.4-adjusted medium containing 10 ng/ml TNF-α. DEX does not affect serine 536 phosphorylation of NF-κB p65 in all cases, pH 6.3, pH 7.4, and TNF-α (pH 7.4). Representative data of 3 independent experiments are shown. **d** Binding of NF-κB p65 to its consensus DNA sequence is significantly increased at 60 min after pH 6.3- or TNF-α-stimulation compared with pH 7.4-stimulation (*P* < 0.01). DEX (1 μM) does not substantially affect the binding of NF-κB p65 to its consensus DNA sequence in all cases, pH 6.3, pH 7.4, and TNF-α (pH 7.4). Data are expressed as percentages of absorbance at 450 nm (OD450) of pH 6.3-stimulated ASMCs without DEX (mean ± SEM, *n* = 5; NS, not significant)

Serine 536 phosphorylation of NF-κB p65 was investigated in ASMCs. TNF-α (10 ng/mL)-stimulated ASMCs were used as a positive control of NF-κB p65 phosphorylation. The amount of phosphorylated NF-κB p65 was markedly increased at 30–60 min after addition of 10 ng/mL TNF-α. It was also slightly increased at 30–60 min in pH 6.3-stimulated ASMCs, but not in pH 7.4-stimulated ASMCs (Fig. 6a). In the presence of BAY11–7082, phosphorylation of NF-κB p65 was almost completely inhibited with stimulation in all cases, pH 6.3, pH 7.4, and TNF-α (Fig. 6b). Next, whether DEX (1 μM) affected serine 536 phosphorylation of NF-κB p65 was examined in ASMCs. Thirty-min preincubation with DEX (1 μM) did not change the amount of phosphorylated p65 in all cases, pH 6.3, pH 7.4, and TNF-α-stimulated ASMCs (Fig. 6c). The binding of NF-κB p65 to its consensus DNA sequence (5’-GGGACTTTCC-3′) was analyzed. The binding of NF-κB p65 to DNA was increased in either pH 6.3 or TNF-α (10 ng/mL)-stimulated ASMCs compared with that in the cells incubated in pH 7.4-adjusted medium (*P* < 0.01). The increase of DNA binding was smaller in acidic pH-stimulated ASMCs than in TNF-α-stimulated cells. DEX (1 μM) did not affect the binding of NF-κB p65 to its consensus DNA sequence in all cases, pH 6.3, pH 7.4, and TNF-α (10 ng/mL) (Fig. 6d).

## Discussion

Hypertrophy and hyperplasia of airway smooth muscle cells (ASMCs) are characteristic pathological findings of severe asthma [1, 39]. ASMCs are increasingly recognized as an important source of inflammatory cytokines and chemokines, as well as the effector cells of bronchoconstriction [2–8, 40]. Bronchial thermoplasty has recently been introduced as a nonpharmacological therapy for moderate-to-severe asthma patients who are uncontrolled despite optimal medical therapy. This treatment could reduce exacerbations and the emergency room visit rate, resulting in improved quality of life [41–43]. Although the mechanism of action is incompletely understood, it has been suggested that bronchial thermoplasty works by reducing ASMCs [44]. Reduction of ASMCs may decrease the secretion of cytokines and chemokines from ASMCs in patients with severe asthma. Interestingly, it has been demonstrated that increased expressions of CXCL8 and eotaxin in ASMCs, as well as an increase of airway smooth muscle area, are seen in patients with severe asthma compared with those with moderate asthma [45].

CXCL8 is secreted by ASMCs following various stimuli, such as TNF-α and cigarette smoke [7, 26–34]. Our previous study showed that extracellular acidification induced ASMCs to generate IL-6 and CTGF via OGR1 [18, 19]. In the present study, the ASMCs, in which the expression of OGR1 was decreased by siRNA targeted for OGR1, released less CXCL8 in response to extracellular pH 6.3 than controlled ASMCs, suggesting that CXCL8 is also generated and released through OGR1-mediated intracellular signal transduction in acidic pH-stimulated ASMCs. The amount of CXCL8 released from pH 6.3-stimulated cells was substantial and about 20–25% of that from TNF-α-stimulated ASMCs. Because we have previously shown that IL-6 release is inhibited by a MEK1/2 inhibitor and a p38 MAPK inhibitor in acidic pH-stimulated ASMCs, the effects of these kinase inhibitors on CXCL8 release in acidic pH-stimulated cells were examined. A MEK1/2 inhibitor, U0126, but not a p38 MAPK inhibitor, SB203580, inhibited CXCL8 release in acidic pH-stimulated ASMCs, suggesting that p38 MAPK activation is not always required for CXCL8 production in ASMCs. In fact, it has been reported that MEK1/2-ERK1/2 activation is required, but p38 MAPK activation is not, for CXCL8 production in poly(I:C)-stimulated human fetal ASMCs [46] or heat shock protein 22-stimulated aortic smooth muscle cells [47]. CXCL8 gene transcription seems to be regulated by transcription factors, such as NF-κB, AP-1, or C/EBP, because the binding sites of these transcription factors are present in the CXCL8 promoter region [47, 48]. Among them, NF-κB is generally involved in CXCL8 gene transcription in many kinds of cells [49–51]. In this study, serine 536 phosphorylation of NF-κB p65 was increased after acidic pH stimulation, in contrast to our previous study [18]. This discrepancy may depend on individual differences of the primary ASMCs used in the present study. Phosphorylation at Ser536 on the p65 subunit of NF-κB is mediated by IKK during LPS stimulation. Ser536 phosphorylation is responsible for the recruitment of coactivators such as p300, promoting the transcriptional activation of NF-κB and the subsequent production of inflammatory cytokines [52–54]. Interestingly, BAY 11–7082, an inhibitor of the NF-κB pathway, almost completely inhibited the serine 536 phosphorylation of NF-κB p65 and acidic pH-stimulated CXCL8 production in ASMCs. CXCL8 secretion from ASMCs in the presence of BAY 11–7082 was less than that in the pH 7.4-adjusted medium without any stimulation. These results suggest that a certain level of NF-κB activity may be essential for CXCL8 production in ASMCs, and that NF-κB and ERK1/2 are major regulators of CXCL8 production in ASMCs induced by acidic pH stimulation, as well as other stimuli [30, 46, 55, 56]. Recently, it was reported that OGR1-mediated NF-κB activation is required for acidic pH-induced CXCL8 production in a human pancreatic β-cell line [51]. This also indicates that NF-κB activity is essential for OGR1-mediated CXCL8 production.

OGR1 has been shown to couple to Gq/11 protein in ASMCs [17, 18]. It is expected that phospholipase C (PLC) is activated and mediates the hydrolysis of membrane inositol phospholipids to diacylglycerol (DAG) and inositol 1,4,5-trisphosphate, which in turn triggers the release of calcium from intracellular stores following Gq/11 activation [14, 57, 58]. Because the PKC inhibitor bisindolylmaleimide I inhibited OGR1-mediated CXCL8 secretion in ASMCs, PKC activation following PLC may be involved in the pathway to CXCL8 gene transcription. In the present study, DEX inhibited CXCL8 secretion from acidic pH-stimulated ASMCs in a dose-dependent manner. CXCL8 mRNA expression was also inhibited by DEX, as well as CXCL8 protein. Although DEX partially inhibited CXCL8 release in TNF-α-stimulated ASMCs, the inhibition rate was smaller than that in acidic pH-stimulated cells. Glucocorticoids (GCs) may regulate cytokine gene expression in several ways. The glucocorticoid receptor (GR) influences gene expression by physically interacting with other transcription factors without contacting DNA itself. This mechanism is called transrepression [59]. Activated GRs translocate to the nucleus and bind to coactivators to inhibit histone acetyltransferase activity and recruiting histone deacetylase-2, which reverses histone acetylation, leading to suppression of targeted genes [60, 61]. Although NF-κB activity was essential for CXCL8 secretion of ASMCs, DEX affected neither serine 536 phosphorylation of NF-κB p65 nor binding of NF-κB p65 to its consensus DNA sequence. These results support the possibility that CXCL8 gene transcription is inhibited by DEX at the step after a dimer of p50 and p65 NF-κB proteins binds to a specific NF-κB recognition site. The detailed mechanism of DEX with respect to CXCL8 gene transcription in ASMCs and the reason why the inhibition rate by DEX is different between acidic pH-stimulated cells and TNF-α-stimulated ones should be elucidated by further research.

As a limitation of this research, our experiments were done using ASMCs originated from non-diseased individuals. If CXCL8 secreted via OGR1 is involved in neutrophilic airway inflammation of COPD, the results in this study might support the usefulness of inhaled corticosteroids as well as long acting bronchodilators in pharmacotherapy of COPD. In future, it is very important to investigate whether DEX inhibits OGR1-mediated CXCL8 secretion in ASMCs isolated from patients with steroid-resistant asthma or COPD, because ASMCs of patients with severe asthma seem to be insensitive to corticosteroids [62, 63].

## Conclusions

Taken together, the present data demonstrate for the first time that extracellular acidification induces CXCL8 secretion of ASMCs through OGR1-mediated cellular activation, and that DEX inhibits OGR1-mediated CXCL8 secretion of ASMCs originated from non-diseased individuals.

## Abbreviations

AP-1: Activator protein 1
ASMC: Airway smooth muscle cell
C/EBP: CCAAT/enhancer binding protein
COPD: Chronic obstructive pulmonary disease
CTGF: Connective tissue growth factor
CXCL8: C-X-C motif chemokine ligand 8
CXCR2: C-X-C motif chemokine receptor 2
DEX: Dexamethasone
DMSO: Dimethyl sulfoxide
ERK: Extracellular signal-regulated protein kinase
EtOH: Ethanol
GC: Glucocorticoid
IKK: IκB kinase
IL-6: Interleukin-6
LPS: Lipopolysaccharide
MAPK: Mitogen-activated protein kinase
MEK: MAP/ERK kinase
NF-κB: Nuclear factor-kappa B
OGR1: Ovarian cancer G protein-coupled receptor 1
PKC: Protein kinase C
PLC: Phospholipase C
TNF-α: Tumor necrosis factor-α
